# Chapped Nipples

**Published:** 1854-01

**Authors:** 


*Chapped Nipples.*—Take of olive oil, ℥x.; venice turpentine, ℥ij.; yellow wax, ℥j.; alkanet root, ℥ss.; boil together, strain, and add balsam of Peru, ℥ijss.; camphor, gr. x.; stir constantly until cold. This is highly esteemed for chapped nipples and broken chillblains.—*From the N. H. Journal of Medicine.*

